# Type 2 diabetes is associated with pulmonary cavitation in men with HIV-TB coinfection

**DOI:** 10.3389/fendo.2026.1635725

**Published:** 2026-01-23

**Authors:** Yongkang Mao, Bennan Zhao, Lijuan Lan, Fengjiao Gao, Xiaoxia Ren, Jingchang Du, Yanfeng Zhu, Dafeng Liu

**Affiliations:** 1 The First Ward of Internal Medicine, Public Health Clinical Center of Chengdu, Chengdu, China; 2 School of Public Health, Chengdu Medical College, Chengdu, China

**Keywords:** correlation, cross-sectional study, hiv/aids, pulmonary cavity, tuberculosis, type 2 diabetes

## Abstract

**Objectives:**

To explore the association between type 2 diabetes mellitus (T2D) and pulmonary cavitation in male with HIV–tuberculosis (TB) coinfection, as well as to quantify the relationships between glycemic indicators [HbA1c and fasting plasma glucose (FPG)] and cavity size. The robustness of these correlations was further validated in a non-HIV TB sample.

**Methods:**

This comparative cross-sectional study based on exposure status included 132 men with HIV–TB and T2D (exposed group) and 131 age-matched men with HIV–TB without T2D (non-exposed group). Multivariable regression models, subgroup analyses, and interaction tests were used to evaluaterelationships and effect modification. A validation cohort of 100 non-HIV TB patients was analyzed using the same analytical framework.

**Results:**

In men coinfected with HIV and TB, T2D was linked to a higher incidence of pulmonary cavitation (adjusted OR = 3.892, 95% CI = 1.895-7.992, P<0.001). HbA1c (B = 1.039, P = 0.049) and FPG (B = 0.869, P<0.001) are positively correlated with cavity size. A notable interaction was detected between T2D and sputum positivity (P<0.001), indicating the greatest incidence of cavitation in sputum-positive T2D patients (OR = 10.492, 95% CI = 3.266–33.711). Consistent results were found in the non-HIV TB group (T2D-related cavitation OR = 4.110, P = 0.014), demonstrating that the effect of T2D is not modified by HIV status.

**Conclusion:**

T2D is a significant risk factor for pulmonary cavitation in males with HIV–TB coinfection, and poor glycemic management is linked with increased cavity size. Sputum-positive patients with T2D represent an exceptionally high-risk subgroup. Incorporating glycemic evaluation and optimal metabolic management into TB care may assist to lower cavitation risk in this population.

## Introduction

The coexistence of type 2 diabetes mellitus (T2D) with HIV and tuberculosis (TB) is a considerable public health challenge, particularly in low and middle income nations (1, 2). Together, these three conditions exert a cumulative effect that substantially amplifies disease burden. Although antiretroviral therapy (ART) has reduced HIV infection into a manageable chronic condition, metabolic complications—particularly T2D—have emerged as significant comorbidities. Given the high burden of TB among people living with HIV, increasing attention has been directed toward interactions between metabolic disorders and TB. Emerging evidence suggests that dysglycemia is linked with TB risk and disease severity (3, 4).

As a principal metabolic illness, T2D might increase the risk of tuberculosis and exacerbate lung tissue damage by causing systemic inflammation and impairing the body’s anti-tuberculosis immune response (5, 6). Higher blood glucose levels may indirectly indicate an increased bacterial load and more severe lung tissue destruction, with pulmonary cavities being a common symptom (7). A recent study found that 54.8% of diabetic individuals had cavities, compared to 27.6% in the control group (8). A tentative link between T2D and pulmonary cavities has been found in those co-infected with HIV and TB, albeit the underlying mechanism is unknown. As a critical sign of severe tuberculosis, pulmonary cavities are related with treatment failure, higher recurrence risk, and an increased disease transmission probability (9, 10). However, there is insufficient evidence to suggest that the impact of T2D on pulmonary cavities in people co-infected with HIV and TB is largely unrelated to measured immunological indicators – a fundamental concern motivating this research.Notably, epidemiological statistics show large gender discrepancies in HIV-TB co-infected populations, this also justifies the focus of this study on the male population. A cross-sectional research of 1,261 patients co-infected with HIV and tuberculosis in India discovered that the prevalence rate among males was as high as 77.69% (11). A large-scale cohort research in Iran reveals that males account for 84.82% of those affected. Male patients have faster clinical progression and a higher risk of severe disease, with mortality rates around 7 percentage points higher than those of females (12, 13). Previous research has found that males are a risk factor for the creation of tuberculosis cavities, and males have a worse prognosis than females in the context of TB (14). Despite rising understanding of the syndemic, considerable information gaps remain. To begin, the majority of prior research has focused on broad TB populations or non-gender analysis. There is insufficient research on the particular connection between T2D and pulmonary cavities in HIV-infected men. Second, previous analyses could not reliably account for major HIV-related variables including CD4+ T cell count and HIV viral load, making it difficult to determine the association between T2D and pulmonary cavities.This Comparative cross-sectional study seeks to fill these gaps by looking into the link between type 2 diabetes and pulmonary cavities in males with HIV-TB co-infection. The study will provide evidence for the integrated clinical management of this high-risk population.

## Materials and methods

### Data sources and study population

**Figure 1:**
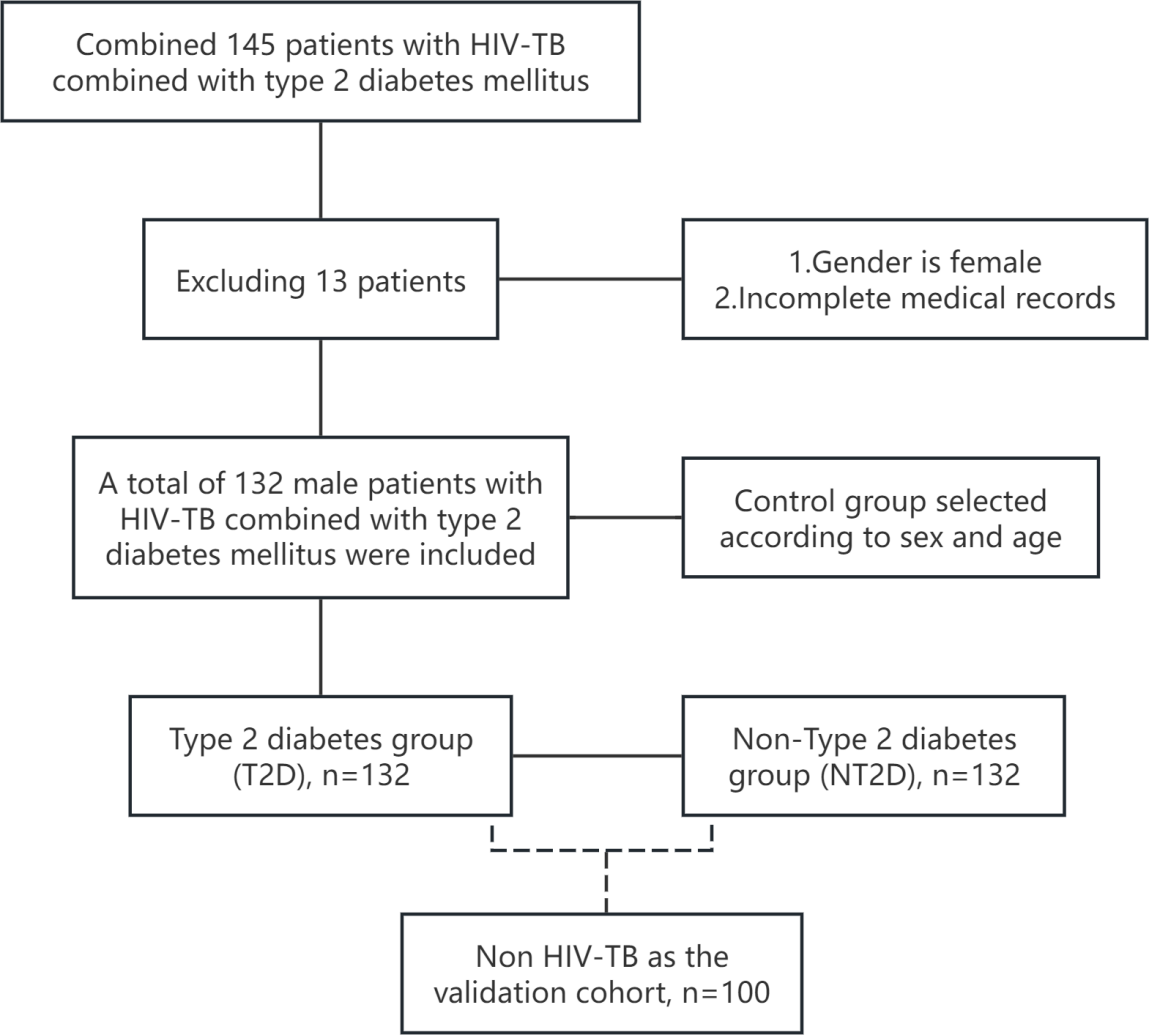
Inclusion process for case and control groups.

This observational comparative cross-sectional study based on exposure status included male patients with HIV, TB, and T2D as the exposed group (n = 132), and age-matched male patients without T2D ((non-type 2 diabetes mellitus, NT2D) group, n = 131) who were diagnosed with HIV and TB. Participants in the non-exposed group were identified from HIV–TB patients treated at the same clinical center during the same study period (January 2017 to June 2024) and were individually matched to cases in a 1:1 ratio based on age (± 3 years). A slight imbalance in sample size between the two groups (132 cases vs. 131 controls) occurred because no eligible control meeting all matching criteria was available for the final enrolled case (**Figure 1**). Age was used as the matching variable, while other clinical factors were accounted for in subsequent regression, subgroup, and interaction analyses.

The diagnostic criteria for the diseases are as follows: 1.HIV/AIDS: Diagnosed using the Chinese Guidelines for HIV/AIDS Diagnosis and Management (15). 2. Tuberculosis using the Chinese Guidelines for TB Diagnosis and Management (16, 17). 3. T2D is diagnosed by endocrinologists using established criteria (fasting plasma glucose (FPG) ≥7.0 mmol/L, HbA1c≥6.5%, or documented antidiabetic medication) (18, 19).

T2D group Inclusion criteria (1): Male adults aged 18 years or older (2). Complete medical records, including HIV/TB/T2D test results and treatment histories.

T2D group Exclusion criteria include current or recent immunosuppressive therapy within 6 months (e.g., corticosteroids >10 mg prednisone-equivalent/day, chemotherapy) (2). Type 1 diabetes (T1D, due to its distinct pathophysiology compared with T2D. e.g., autoimmune origin vs. insulin resistance-related pathogenesis), gestational diabetes, or secondary diabetes (such as pancreatic origin) (3). Incomplete laboratory or imaging data needed for co-diagnosis validation.

To validate the robustness of the core findings, this study included 100 non-HIV-TB patients as a validation cohort and compared baseline characteristics with 263 HIV-TB co-infected patients (HIV group). Results are presented in **Supplementary Table S1**.

### Measurements

Demographic, metabolic, immunologic, and clinical data were extracted systematically from electronic medical records using established case report forms, and all data were collected at baseline before the initiation of TB treatment. The operational definitions met international consensus criteria (Laboratory Instruments and Corresponding Reagents see **Supplementary Table S2** for details):

#### Demographic characteristics

Marital status: Categorized as married, unmarried, divorced, or widowed based on self-report and medical records.

Smoking status: Defined as smoker (cumulative smoking ≥100 cigarettes or current smoking) or non-smoker (cumulative smoking <100 cigarettes and no current smoking).

Alcohol consumption: Classified as drinker (drinking ≥1 time/week for ≥3 months) or non-drinker (never drinking or drinking <1 time/week).

Metabolic parameters: All study participants (both the T2D and NT2D groups) were tested for glycemia-related indicators at baseline.

(1) HbA1c: Measured via ion-exchange high-performance liquid chromatography (reference range 4.0–6.0%). For T2D patients, glycemic control status was defined per the National Guidelines for the Prevention and Control of Diabetes in Primary Care (2022), poor glycemic control (HbA1c ≥7.0%) and good glycemic control (HbA1c <7.0%) (19) (2). FPG: Plasma glucose (mmol/L) after ≥8-hour fasting, analyzed by hexokinase method.

#### Hematological profiles

(1) Complete blood counts (Sysmex XN-9100): White blood cell count (WBC; ×10^9^/L); Red blood cell count (RBC; ×10¹²/L); Platelet count (PLT; ×10^9^/L).

(2) Inflammatory Biomarkers: C-reactive protein (CRP): Quantified by particle-enhanced immunoturbidimetric assay; Interleukin-6 (IL-6): Assessed using chemiluminescence immunoassay (sensitivity 2 pg/mL).

#### Lymphocyte subsets

(1) CD3^+^ T cells (total T lymphocytes): Quantified via four-color flow cytometry following the Chinese Guidelines for HIV/AIDS Diagnosis and Management (2021 edition) (15), with absolute counts (cells/μL) calculated using BD TruCOUNT™ beads.

(2) CD4^+^ T helper cells: Absolute counts (cells/μL) determined by four-color flow cytometry (same as above) and standardized with BD TruCOUNT™ beads for accuracy.

(3) CD8^+^ cytotoxic T cells: Absolute counts (cells/μL) measured via four-color flow cytometry (refer to CD3^+^ T cell detection protocol) and verified using BD TruCOUNT™ beads.

(4) Absolute lymphocyte count (LYC): Calculated from differential counts (standard hematology analyzer). Results expressed as absolute counts (cells/μL) using TruCOUNT™ beads.

Quality control for flow cytometry was performed daily using BD Cytometer Setup and Tracking beads. The inter-assay coefficient of variation (CV) was less than 5% for all T lymphocyte subsets, ensuring reliable detection.

#### Tuberculosis cavities assessment

Defined as ≥1 cavity with wall thickness >2 mm on high-resolution chest CT, independently reviewed by two radiologists blinded to clinical status. The size of pulmonary cavities was measured on cross-sectional chest CT images and expressed as the cavity’s maximum diameter on the largest plane in millimetres (mm). For patients with multiple cavities, the maximum diameter of the largest cavity was used as the indicator of that patient’s cavity size.

#### Sputum positive

Acid-fast bacilli detected on at least two sputum smears or growth of Mycobacterium tuberculosis in Löwenstein-Jensen medium or Mycobacteria Growth Indicator Tube system.

#### TB dissemination

(1) Intra pulmonary TB: Confirmed by ≥1 of the following: (a) acid-fast bacilli (AFB) positivity on ≥2 sputum smears; (b) Mycobacterium tuberculosis (MTB) growth in Löwenstein-Jensen (LJ) medium or Mycobacteria Growth Indicator Tube (MGIT) system; (c) Xpert MTB/RIF assay positivity plus chest CT evidence of TB (e.g., cavitation, infiltrates).

(2) Intra and extra pulmonary TB: A diagnosis is confirmed if the criteria for intra-pulmonary TB are met and at least one of the following conditions is satisfied: (a) MTB growth in culture of extrapulmonary specimens (e.g., lymph node aspirate, pleural fluid, bone marrow); (b) histopathological evidence of caseous necrosis plus MTB DNA detection via PCR; (c) clinical symptoms consistent with EPTB (e.g., lymphadenopathy, pleural effusion) plus response to anti-TB therapy (symptom resolution within 8 weeks). This study conducted subgroup analyses by diagnostic method and incorporated diagnostic methods into regression models to systematically evaluate the potential confounding effects of differences in diagnostic methods in pulmonary tuberculosis. This dual validation approach ensured the robustness of the conclusion linking T2D to pulmonary cavitation, as shown in **Supplementary Table S3** and **S4**.

#### ART exposure

(1) ART-treated: Initiation of WHO-recommended first-line antiretroviral therapy (ART) regimen (two nucleoside reverse transcriptase inhibitors [NRTIs] plus one non-nucleoside reverse transcriptase inhibitor [NNRTI]/integrase strand transfer inhibitor [INSTI]) for ≥6 months, with pharmacy-verified adherence ≥90%. Key drugs included tenofovir (TDF/TAF), lamivudine (3TC), efavirenz (EFV), and dolutegravir (DTG).

(2) Non-ART: No documented exposure to antiretroviral drugs prior to study enrollment.

HIV viral load: Quantified via real-time RT-PCR with a lower limit of detection (LOD) of 50 copies/mL. Results were categorized as undetectable (≤50 copies/mL) or detectable (>50 copies/mL).

In addition, data on disease duration, treatment duration, comorbidities, and hypoglycemic medication use among patients with T2D were provided as **Supplementary Material**. Detailed definitions and diagnostic or measurement criteria for each supplementary variable, as well as medication-related statistics, are available in Supplementary Material 4 (Definition and Measurement Methods of Disease Duration and Comorbidity Assessment) and **Supplementary Table S5** (Medication-Related Information in Patients with T2D).

### Statistical analysis

Continuous data are presented as Mean ± SD (normal distribution) or M (Q1,Q3) (non-normal distribution), whereas categorical variables are reported as numbers and percentages (%).

Intergroup differences were assessed using appropriate statistical techniques, such as Chi-square tests for categorical variables and t-tests or Mann-Whitney U test (does not satisfy normality) for continuous data.

The Shapiro–Wilk test was applied to assess the normality of continuous variables. Univariate regression analyses were performed to examine the associations between each independent variable and two key outcomes related to pulmonary cavitation: For pulmonary cavitation (a binary outcome defined as present or absent), univariate binary logistic regression analysis was used to calculate odds ratios (OR) and 95% confidence intervals (CI), which quantified the magnitude of the association between each independent variable and the risk of pulmonary cavitation; For cavity size, univariate linear regression analysis was conducted to compute regression coefficients (B) and 95%CI, which reflected the extent of change in cavity size associated with per unit change in the independent variable. Variables with biological plausibility and/or significant associations (P < 0.05) in the univariate regression analyses were selected for subsequent multivariate regression analyses, aiming to adjust for potential confounders and verify the effects of T2D and glycemic indicators on pulmonary cavitation and cavity size. Receiver operating characteristic (ROC) curves with area under the curve (AUC) and 95% CI were created to assess the diagnostic value of pulmonary cavities. Statistical analyses were carried out using SPSS 27.0, and graphics were prepared with GraphPad Prism 10.2.0.

A two-tailed P-value <0.05 indicated statistical significance.

### Multivariate regression analysis

Regression models were employed to analyze two sets of key associations: 1) the relationship between T2D and pulmonary cavities, as well as between HbA1c and FPG levels and the size of pulmonary cavities; 2) the relationship between sputum bacterial positivity and pulmonary cavities. Parallel three-model frameworks were developed for both T2D and sputum bacterial positivity to assess their respective influences on pulmonary cavities, with consistent covariate adjustment strategies— the only difference between the two frameworks lies in the core independent variable (T2D for the first framework, sputum bacterial positivity for the second framework), while the other covariates (including the non-core one of the two key variables) were uniformly incorporated in the full-adjustment model. For the analysis of pulmonary cavity size, HbA1c and FPG were used as primary independent variables, adopting the same covariate adjustment criteria as the above two frameworks.

#### Regression framework with T2D as the core independent variable

Three models were constructed to evaluate the association between T2D and pulmonary cavities: Model 1 included T2D (primary independent variable) and HbA1c/FPG (for cavity size analysis). Model 2 introduced age as a covariate to Model 1. Model 3 further incorporated all candidate variables that showed statistically significant associations in univariate regression analyses (based on Model 2), including current smoking status, sputum bacterial positivity/negativity, CRP, WBC count, CD4^+^ T cell count, and fatty liver disease. CD3^+^ T cells, CD8^+^ T cells, and LYC, though significantly associated with pulmonary cavities in univariate regression, were excluded due to collinearity with CD4^+^ T cells to ensure model stability.

#### Regression framework with sputum bacterial positivity as the core independent variable

Consistent with the T2D-based framework, three parallel models were constructed to evaluate the effect of sputum bacterial positivity on pulmonary cavities: Model 1 included sputum bacterial positivity (primary independent variable) and HbA1c/FPG (for cavity size analysis). Model 2 introduced age as a covariate to Model 1. Model 3 further incorporated the same set of variables with statistically significant associations in univariate regression analyses (as in the T2D-based Model 3), including current smoking status, T2D, CRP, WBC count, CD4^+^ T cell count, and fatty liver disease. Similarly, CD3^+^ T cells, CD8^+^ T cells, and LYC were excluded due to collinearity with CD4^+^ T cells to avoid multicollinearity-induced bias.

To assess the generalizability of the core findings to the non-HIV cohort, the same analytical strategy utilized for the HIV-positive cohort was employed, resulting in the construction of three models. Model 1 utilized T2D and HbA1c/FPG as primary independent variables in binary logistic regression and multiple linear regression, respectively. Model 2 included age as a covariate in Model 1. Model 3 incorporated the distribution of tuberculosis, sputum bacterial positivity/negativity, CRP, and WBC count as covariates in addition to Model 2.

Furthermore, to evaluate the potential impact of HIV status on core associations, distinct regression models were developed for the combined non-HIV group representing the total population. Three regression models were developed for the HIV status variable: Model 1 included T2D as the primary independent variable and HIV status as a covariate; Model 2 incorporated age as an additional covariate to Model 1; and Model 3 included the distribution of tuberculosis, sputum bacterial positivity/negativity, CRP, and WBC levels as covariates to Model 2.

### Subgroup analysis

To validate the robustness of the association between T2D and pulmonary cavities across different subgroups, and to exclude potential stratification effects, we performed a subgroup analysis based on the following stratifications: (1) age (divided into <50 years and ≥50 years groups); (2) CD4+ cell count (divided into <200 and ≥200 cells/μL groups); (3) HIV viral load (divided into <50 and ≥50 copies/mL groups); (4) ART status (divided into yes and no); (5) HIV (divided into yes and no); (6) sputum positive (divided into yes and no); (7) TB distribution (divided into Intrapulmonary and Intra- and extra-pulmonary groups); (8) WBC level (divided based on the median value into <5.6 ×10^9^/L and ≥5.6 ×10^9^/L); and (9) CRP level (divided based on the median value into <46.7 mg/L and ≥46.7 mg/L; (10) current smoking status (divided into smoker and non-smoker group). Each subgroup employed a binary logistic regression model consistent with the primary analysis to assess the association between T2D and pulmonary cavitation. The analysis output the OR, 95% CI and P-value for each subgroup. The robustness of the conclusions was evaluated by comparing the consistency of the OR and P-values across subgroups.

An interaction term was created for each stratification variable and T2D, and the interaction P-value was reported. A P-value for the interaction below 0.05 indicates that the stratification variable significantly affects the core association between T2D and pulmonary cavities. A P-value exceeding 0.05 indicates an absence of a significant interaction effect, suggesting that the core association remains consistent and robust across various subgroups of the variable.

In addition, we aimed to explore the associations between the above-mentioned stratification factors and pulmonary cavitation in the NT2D population, and to compare with the T2D group to clarify the specificity of the associations. The same stratification criteria as the T2D group were adopted, and a binary logistic regression model with identical adjustment factors was used for each stratum. Here, each stratification factor (e.g., sputum positivity, age) was the core independent variable (e.g., sputum positive vs. negative) and pulmonary cavitation as the dependent variable. The output included the OR, 95%CI, and P-value for each stratification factor in the NT2D group, to reflect the strength of the association between these factors and pulmonary cavitation in the absence of T2D.

T2D were stratified into Well-controlled and Poorly controlled subgroups. Size of cavity was modeled as a continuous dependent variable, and the associations of HbA1c and FPG with size of cavity were assessed separately using linear regression.

### Ethics statement

The researchers collected data from the participants through electronic medical record information while strictly adhering to the ethical guidelines set forth in the Declaration of Helsinki. Chengdu Public Health Clinical Medicine (YJ-K2025-34-01) approved the study and all participants gave informed consent.

## Results

### Comparison of sociodemographic characteristics, comorbidities, and clinical features between patients with T2D and NT2D

As shown in **Table 1**, there were no significant differences in sociodemographic characteristics (age, marital status, current smoking status, alcohol use), comorbidities [Chronic Obstructive Pulmonary Disease (COPD), hyperlipidemia], and clinical characteristics (PLT, CRP, IL-6 and WBC) between the T2D group and the NT2D group (all P > 0.05). Among comorbidities, the proportions of hypertension [25.8% (34/132) vs. 9.9% (13/131); χ² = 11.231, P < 0.001], fatty liver disease [13.6% (18/132) vs. 3.8% (5/131); χ² = 7.944, P = 0.005], and viral hepatitis [19.7% (26/132) vs. 9.9% (13/131); χ² = 4.972, P = 0.026] were significantly higher in the T2D group than in the NT2D group. Among glucose metabolism-related indicators, the median HbA1c was significantly higher in the T2D group than in the NT2D group [7.80 (6.50, 9.45) vs. 5.90 (5.70, 6.10); Z = -5.051, p < 0.001], as was the mean FPG [10.778 ± 5.599 vs. 6.259 ± 1.908; t = -8.748, p < 0.001]. Among haematological indicators, the median RBC count was slightly higher in the T2D group than in the NT2D group [4.09 (3.26, 4.35) vs. 3.95 (3.60, 4.29, Z = -2.27, P = 0.023). Overall comparability was maintained between the two groups.

**Table 1:** Comparison of sociodemographic characteristics, comorbidities, and clinical features between patients with T2D and NT2D.

Items	T2D [n (%)/Mean±SD/M (Q1,Q3)]	NT2D [n (%)/Mean±SD/M (Q1,Q3)]	χ/t/Z	P
Age (years)	55.300±12.128	55.240±12.073	-0.039	0.969^a^
Marital status			2.183	0.153^c^
Married	93 (70.5)	81 (61.8)		
Unmarried	39 (29.5)	50 (38.2)		
Current smoking status			0.043	0.836^c^
Yes	82 (62.1)	83 (63.4)		
No	50 (37.9)	38 (36.6)		
Alcohol use			1.675	0.196^c^
Yes	72 (54.4)	61(46.6)		
No	60 (45.5)	70 (53.4)		
Hypertension			11.231	<0.001^***c^
Yes	34 (25.8)	13 (9.9)		
No	98 (74.2)	118(90.1)		
Chronic Obstructive Pulmonary Disease			0.397	0.528^c^
Yes	12 (9.1)	15 (11.5)		
No	120 (90.9)	116 (88.5)		
Fatty liver disease			7.944	0.005^**c^
Yes	18 (13.6)	5 (3.8)		
No	114 (86.4)	126 (96.2)		
Hyperlipidemia			0.542	0.462^c^
Yes	10 (7.6)	7 (5.3)		
No	122 (92.4)	124 (94.7)		
Viral hepatitis			4.972	0.026^*c^
Yes	26 (19.7)	13 (9.9)		
No	106 (80.3)	118 (90.1)		
Glucose-lowering therapy			–	–
Yes	77 (58.3)	–		
No	55(41.66)	–		
Glycemic control status			–	–
Well-controlled	36 (27.27)	–		
Poorly controlled	96 (72.72)	–		
Duration of T2D (days)	1825.00 (730.00, 3650.00)	–	–	–
Duration of T2D treatment (days)	1818.00 (363.60, 1638.00)	–	–	–
HbA1c (%)	7.80 (6.50, 9.30)	5.90 (5.70, 6.10)	-5.051	<0.001^***b^
FPG (mmol/L)	10.778±5.599	6.259±1.908	-8.748	<0.001^***a^
WBC (×10^9^/L)	5.18 (3.84, 7.92)	4.82 (3.70, 6.12)	-0.562	0.574^b^
RBC (×10¹²/L)	4.09 (3.26, 4.35)	3.95 (3.60, 4.29)	-2.27	0.023^*b^
PLT (×10^9^/L)	205.017±129.680	198.420±96.422	-0.468	0.64^a^
CRP (mg/L)	54.39 (10.21, 101.76)	49.52 (6.00, 74.04)	-0.925	0.355^b^
IL-6 (ng/L)	27.12 (6.78, 94.71)	28.34 (12.25, 49.30)	-1.138	0.253^b^

T2D, Type 2 diabetes mellitus; NT2D, Non-type 2 diabetes mellitus; HbA1c, Glycated hemoglobin A1c; FPG, Fasting plasma glucose; WBC, White blood cell count; RBC, Red blood cell count; PLT, Platelet count= . * indicates P < 0.05, ** indicates P < 0.01, *** indicates P < 0.001; Mean±SD = Mean ± standard deviation; M (Q1, Q3) = Median (first quartile, third quartile); a denotes the use of an t-test, b denotes the use of the Mann-Whitney U test, c denotes the use of Chi-square test.

Additionally, 58.30% (77/132) of patients in the T2D group received glucose-lowering therapy, with a median T2D duration of 1825.00 (730.00, 3650.00) days and a median glucose-lowering treatment duration of 1818.00 (363.60, 1638.00) days.

### Comparison of HIV/TB-related indicators between T2D and NT2D

**Figure 2:**
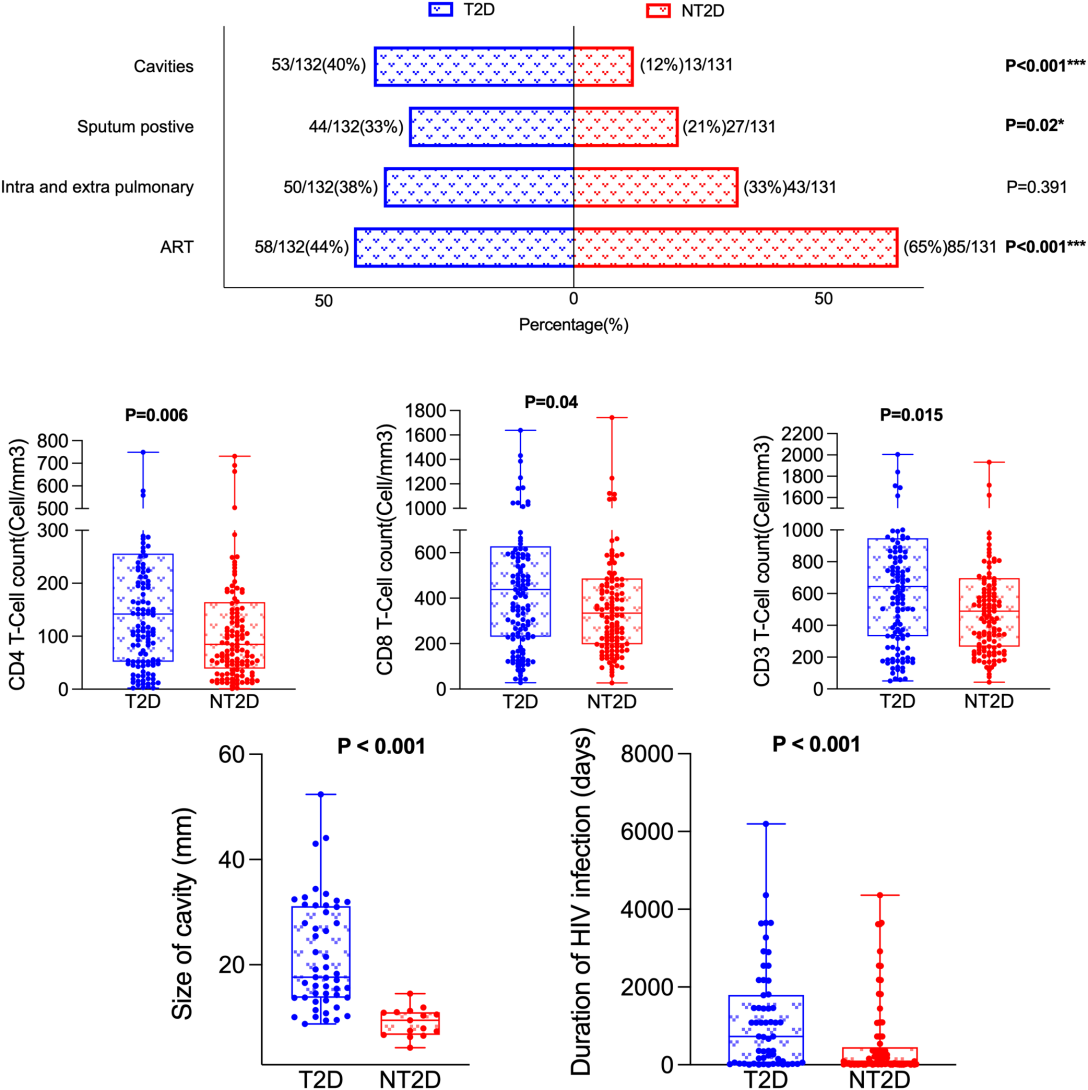
Comparison of HIV-TB-related indicators between T2D and NT2D. T2D, Type 2 diabetes mellitus; NT2D, Non-type 2 diabetes mellitus; ART, Antiretroviral therapy.

**Table 2** and **Figure 2** shows that the incidence of pulmonary cavities (53/132 vs 16/131, χ² = 26.518, P < 0.001) and sputum bacterial positivity (44/132 vs 27/131, χ² = 5.4, P = 0.02) were significantly higher in the T2D group than in the NT2D group. The cavity sizes were also significantly larger in the T2D group [18.27 (13.74, 31.14) vs 9.92 (7.39, 11.02), Z = -4.889, P < 0.001]. No significant differences were observed in tuberculous lesion distribution or duration of TB disease between the two groups.

**Table 2:** Comparison of HIV-TB related indicators Between T2D and NT2D.

Items	T2D [n (%)/M (Q1,Q3)]	NT2D [n/M (Q1,Q3)]	χ/t/Z	P
Cavities			26.518	<0.001^***c^
Yes	53 (40.2)	16 (12.2)		
No	79 (59.8)	115 (87.8)		
Sputum Postive			5.4	0.02^*c^
Yes	44 (33.3)	27 (20.6)		
No	88 (66.6)	104 (79.4)		
Distribution			0.735	0.391^c^
Intrapulmonary	82 (62.1)	88 (67.2)		
Intra- and extra-pulmonary	50 (37.9)	43 (32.8)		
ART			11.628	<0.001^***c^
Yes	58 (43.9)	85 (67.2)		
No	74 (56.1)	46 (32.8)		
Duration of TB disease (days)	30.00 (15.00, 60.00)	30.00 (10.00, 60.00)	-0.135	0.893^b^
Size of cavity (mm)	18.27 (13.74, 31.14)	9.92 (7.39,11.02)	-4.889	<0.001^***b^
Duration of HIV infection (days)	730.00 (75.00, 2007.00)	210.00 (30.00, 730.00)	-1.206	0.228^b^
Duration of ART use (days)	728.00 (44.00, 1799.00)	90.00 (16.50, 452.50)	-3.305	<0.001^***b^
CD3+T Cell(cells/μL)	644.00 (332.00, 948.00)	490.00 (265.50, 697.75)	-2.439	0.015^*b^
CD4+T Cell(cells/μL)	142.00(52.00, 256.00)	84.50 (39.00, 164.25)	-2.733	0.006^**b^
CD8+T Cell(cells/μL)	438.00 (230.00, 628.50)	334.00 (197.00, 487.25)	-2.049	0.04^*b^
CD4:CD8	0.34 (0.19, 0.60)	0.22 (0.08, 0.60)	-1.667	0.096^b^
LYC(10^9^/L)	817.00 (453.50, 1237.50)	666.50 (430.25, 935.75)	-1.943	0.052^b^
HIV viral load (copies/mL)	12200.00 (40.00, 182000.00)	870.00 (40.00, 215000.00)	-1.196	0.232^b^

T2D, Type 2 diabetes mellitus; NT2D, Non-type 2 diabetes mellitus; LYC, Absolute lymphocyte count; ART, Antiretroviral therapy; TB, Tuberculosis.* indicates P < 0.05, ** indicates P < 0.01, *** indicates P < 0.001; n = Number of cases; M (Q1, Q3) = Median (first quartile, third quartile); b denotes the use of the Mann-Whitney U test, c denotes the use of Chi-square test.

Among HIV/ART-related indicators, the rate of ART treatment in the T2D group was significantly lower (58/132 vs 85/131, χ² = 11.628, P < 0.001) while the median duration of ART use was significantly longer [728.00 (44.00, 1799.00) vs 90.00 (16.50, 452.50) days, Z = -3.305, P < 0.001] compared with the NT2D group. The median values for CD3, CD4 and CD8 were also significantly different between the two groups (Z = -2.439; P = 0.015; Z = -2.733; P = 0.006; Z = -2.733; P = 0.006, Z = -2.049, P = 0.04 respectively). No significant differences were observed in duration of HIV infection, HIV viral load, the CD4:CD8 ratio or LYC counts (all P > 0.05).

### Univariate regression analysis of pulmonary cavitation and its size with key clinical, metabolic and immune indicators

**Table 3** shows that T2D (OR = 4.82, 95% CI: 2.57–9.04), FPG (OR = 1.21, 95% CI: 1.13–1.30), and sputum bacterial positivity (OR = 4.96, 95% CI: 2.73–9.01) were associated with an increased risk of pulmonary cavitation (all P < 0.001). In contrast, HbA1c was not significantly associated with cavitation risk (OR = 1.06, 95% CI: 0.96–1.18, P = 0.233). Other inflammatory and immune-related indicators showed weaker but statistically significant associations (**Table 3**).

**Table 3:** Univariate regression analysis of pulmonary cavitation and its size with key clinical, metabolic and immune indicators.

	Cavities	Size of cavitiy
Items	OR (95%CI) P	B (95%CI) P
T2D	4.822 (2.573, 9.037) <0.001	12.569 (7.137, 18.002) <0.001
HbA1c	1.064 (0.961, 1.178) 0.233	1.277 (0.386, 2.168) 0.006
FPG	1.208 (1.127, 1.296) <0.001	0.898 (0.554, 1.241) <0.001
Sputum Postive	4.956 (2.727, 9.008) <0.001	4.534 (-0.681, 9.749) 0.087
CRP	1.006 (1.001, 1.010) 0.019	0.008 (-0.043, 0.059) 0.309
WBC	1.134 (1.051, 1.223) 0.001	-0.105 (-0.686, 0.475) 0.718
CD3	1.001 (1.000, 1.002) 0.004	0.007 (0.000, 0.014) 0.038
CD4	1.002 (1.000, 1.004) 0.025	0.019 (0.002, 0.036) 0.025
CD8	1.001 (1.000, 1.002) 0.010	0.007 (-0.003, 0.016) 0.153
LYC	1.001 (1.000, 1.001) 0.001	0.005 (0.000, 0.010) 0.055
Fatty liver disease	2.876 (1.205, 6.865) 0.017	0.757 (-6.314, 7.829) 0.831

The outcome variable "Cavities" was a binary variable (present/absent). Binary logistic regression analysis was performed, and the results were expressed as odds ratios (OR) with 95% confidence intervals (95% CI). An OR > 1 indicated that the indicator was associated with an increased risk of cavity formation; The outcome variable "Size of cavity" was a continuous variable (unit: mm). Multiple linear regression analysis was conducted, and the results were presented as regression coefficients (B) with 95% CI. A B > 0 suggested that the indicator was correlated with an increase in cavity size. T2D, Type 2 diabetes mellitus; HbA1c, Glycated hemoglobin A1c; FPG, Fasting plasma glucose; CRP, C-reactive protein; WBC, White blood cell count; CD3^+^T Cell, CD3-positive T lymphocyte; CD4^+^T Cell, CD4-positive T helper lymphocyte; CD8^+^T Cell, CD8-positive cytotoxic T lymphocyte; LYC, Absolute lymphocyte count.

Regarding cavity size, T2D (B = 12.57, 95% CI: 7.14–18.00) and FPG (B = 0.90, 95% CI: 0.55–1.24) exhibited particularly strong positive associations (both P < 0.001), while HbA1c (B = 1.28, 95% CI: 0.39–2.17), CD3^+^ T cell count (B = 0.007, 95% CI: 0.000–0.014), and CD4^+^ T cell count (B = 0.019, 95% CI: 0.002–0.036) were also significantly associated. Sputum bacterial positivity and other variables were not significantly related to cavity size.

Variables not shown in the table, including the CD4:CD8 ratio, IL-6, ART status, age, sociodemographic factors, comorbidities, disease duration, and treatment conditions, showed no significant association with the presence or size of pulmonary cavities (all P > 0.05).

### Regression analysis of T2D/HbA1c/FPG and cavitation

**Table 4** shows that in the HIV+ cohort, after adjusting for confounding factors, T2D remained significantly associated with an increased risk of pulmonary cavitation (Model 3: OR = 3.892, 95% CI = 1.895-7.992, P<0.001); Sputum positive (Model 3: OR = 3.810, 95% CI = 1.893, 7.668, P<0.001); HbA1c (Model 3: B = 1.039, P = 0.049) and FPG (Model 3: B = 0.869, P<0.001) were both significantly positively correlated with cavity size. The results suggest that the presence of T2D significantly increases the risk of pulmonary cavitation, with larger cavities being associated with higher blood glucose levels.

**Table 4:** Regression analysis of T2D, HbA1c and FPG with pulmonary cavitation in HIV-TB coinfected men.

Items	Model1 OR/B (95%CI) P	Model2 OR/B (95%CI) P	Model3 OR/B (95%CI) P
T2D and Pulmonary Cavities			
NT2D	Ref	Ref	Ref
T2D	4.822 (2.573, 9.037) <0.001	4.905 (2.607, 9.229) <0.001	3.892 (1.895, 7.992) <0.001
Sputum postive and Pulmonary Cavities			
Sputum negative	Ref	Ref	Ref
Sputum postive	4.956 (2.727, 9.008) <0.001	4.890 (2.685, 8.906) <0.001	3.810 (1.893, 7.668) <0.001
HbA1c and Size of Cavitiy	1.277 (0.386, 2.168) 0.006	1.209 (0.349, 2.069) 0.007	1.039 (0.004, 2.074) 0.049
FPG and Size of Cavitiy	0.898 (0.554, 1,241) <0.001	0.93 (0.604, 1.256) <0.001	0.869 (0.485,1.254) <0.001

T2D, Type 2 diabetes mellitus; NT2D, Non-type 2 diabetes mellitus; HbA1c, Glycated hemoglobin A1c; FPG, Fasting plasma glucose; OR, Odds ratio (for dichotomous dependent variable); B, Regression coefficient (for continuous dependent variable); CI, Confidence interval; Ref, Reference group; OR corresponds to the binary outcome "pulmonary cavitation (yes/no)"; B represents the regression coefficient for the continuous outcome "size of cavity (mm)”; Core independent variable is T2D Model 1: Unadjusted; Model 2: Adjusted for age; Model 3: Adjusted for age, current smoking status, sputum bacterial positivity, C-reactive protein, white blood cell count, CD4^+^ T cell count, and fatty liver disease; Core independent variable is sputum postive Model 1: Unadjusted; Model 2: Adjusted for age; Model 3: Adjusted for age, current smoking status, T2D, C-reactive protein, white blood cell count, CD4^+^ T cell count, and fatty liver disease; NT2D was set as the reference group.

**Table 5** shows that in the non-HIV cohort, after adjusting for confounding factors, T2D remained significantly associated with an increased risk of pulmonary cavitation (Model 3: OR = 4.110, 95% CI = 1.333–12.674, P = 0.014); HbA1c was not significantly correlated with cavity size (Model 3: B = 1.216, P = 0.398), while FPG remained significantly positively correlated with cavity size (Model 3: B = 1.858, 95% CI = 0.533–3.182, P = 0.009).

**Table 5:** Regression analysis of T2D, HbA1c and FPG with pulmonary cavitation in non-HIV TB.

Items	Model1 OR/B (95%CI) P	Model2 OR/B (95%CI) P	Model3 OR/B (95%CI) P
T2D and Pulmonary Cavities
NT2D	Ref	Ref	Ref
T2D	4.519 (1.643, 12.426) 0.003	5.327 (1.846, 15.373) 0.002	4.110 (1.333, 12.674) 0.014
HbA1c and Size of Cavitiy	1.830 (-0.827, 4.487) 0.156	1.069 (-1.556, 3.695) 0.381	1.216 (-1.979, 4.411) 0.398
FPG and Size of Cavitiy	2.254 (1.099, 3.408) <0.001	1.947 (0.756, 3.139) 0.003	1.858 (0.533,3.182) 0.009

T2D, Type 2 diabetes mellitus; NT2D, Non-type 2 diabetes mellitus; HbA1c, Glycated hemoglobin A1c; FPG, Fasting plasma glucose; OR, Odds ratio (for dichotomous dependent variable); B, Regression coefficient (for continuous dependent variable); CI, Confidence interval; Ref, Reference group; OR corresponds to the binary outcome "pulmonary cavitation (yes/no)"; B represents the regression coefficient for the continuous outcome "size of cavity (mm)”; Model 1: Unadjusted; Model 2: Adjusted for age; Model 3: Adjusted for age, distribution of tuberculosis, sputum bacterial positivity/negativity, CRP and WBC count; NT2D was set as the reference group.

**Table 6** shows that in the overall population (HIV+ vs HIV-), the association between T2D and pulmonary cavities was significant and of comparable strength (OR = 4.822 in HIV+ group, OR = 4.519 in HIV- group, both P<0.001), suggesting that HIV status does not modify the core effect of T2D.

**Table 6:** Regression analysis of T2D, HbA1c and FPG with pulmonary cavitation in the total population (HIV-TB coinfected and non-HIV TB).

Items	Model1 OR/B (95%CI) P	Model2 OR/B (95%CI) P	Model3 OR/B (95%CI) P
T2D and Cavities
NT2D	Ref	Ref	Ref
T2D	4.736 (2.780, 8.069) <0.001	4.958 (2.887, 8.515) <0.001	4.244 (2.334, 7.717) <0.001
HbA1c and Size of Cavitiy	1.337 (0.514, 2.160) 0.002	1.340 (0.516, 2.164) 0.002	1.174 (0.0.314, 2.034) 0.008
FPG and Size of Cavitiy	0.968 (0.647, 1,289) <0.001	0.990 (0.669, 1.311) <0.001	0.912 (0.594,1.230) <0.001

T2D, Type 2 diabetes mellitus; NT2D, Non-type 2 diabetes mellitus; HbA1c, Glycated hemoglobin A1c; FPG, Fasting plasma glucose; OR, Odds ratio (for dichotomous dependent variable); B, Regression coefficient (for continuous dependent variable); CI, Confidence interval; Ref, Reference group; OR corresponds to the binary outcome "pulmonary cavitation (yes/no)"; B represents the regression coefficient for the continuous outcome "size of cavity (mm)”; Model 1: Unadjusted; Model 2: Adjusted for age; Model 3: Adjusted for age, distribution of tuberculosis, sputum bacterial positivity/negativity, CRP and WBC count; NT2D was set as the reference group.

### Subgroup analysis and interaction effect

**Figure 3:**
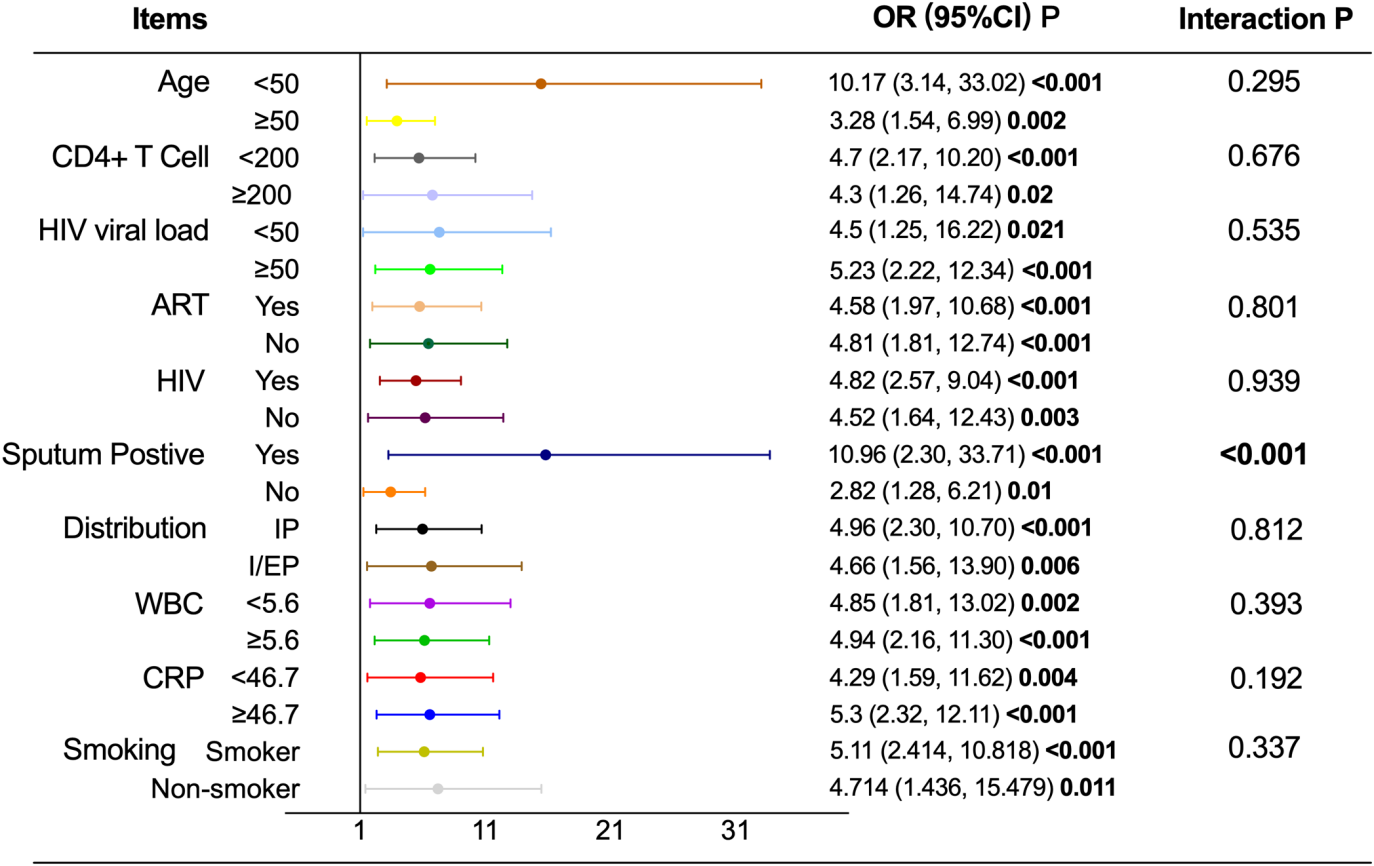
Subgroup analysis and interaction effect of T2D on pulmonary cavitation.T2D, Type 2 diabetes mellitus; NT2D, Non-type 2 diabetes mellitus; OR, Odds ratio; CI, Confidence interval; ART, Antiretroviral therapy; WBC, White blood cell count; CRP, C-reactive protein; Abbreviate intrapulmonary as IP, and intra-pulmonary and extra-pulmonary as I/EP.

**Figure 4:**
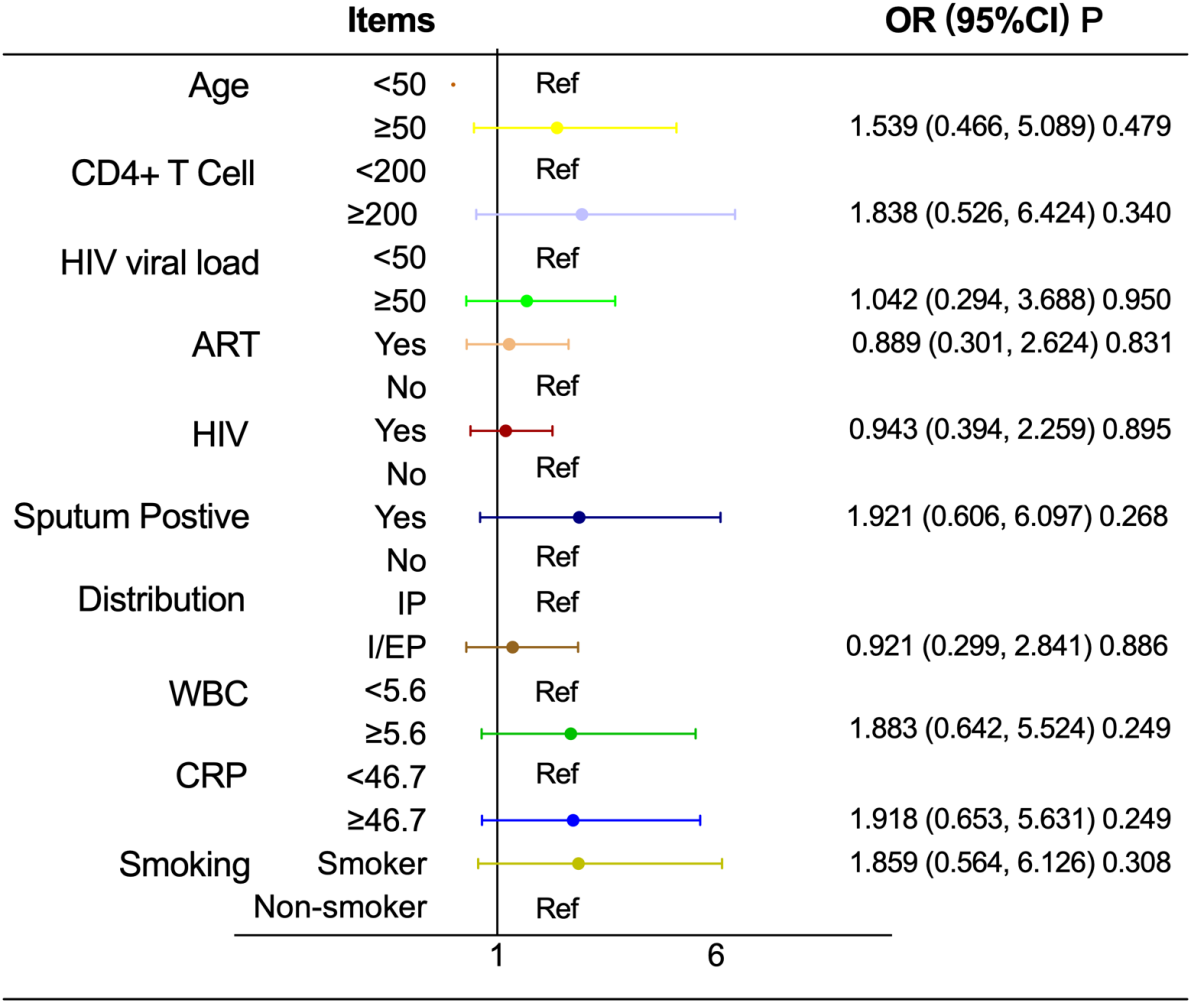
Associations between stratification factors and pulmonary cavitation in HIV-TB coinfected men in NT2D group. OR, Odds ratio; CI, Confidence interval; ART, Antiretroviral therapy; WBC, White blood cell count; CRP, C-reactive protein; Abbreviate intrapulmonary as IP, and intra-pulmonary and extra-pulmonary as I/EP.

As shown in **Table 7** and **Figure 3**, the association between T2D and pulmonary cavitation was significant across all subgroups defined by age, CD4+ count, HIV viral load, ART status, HIV status, pulmonary-only or pulmonary-extrapulmonary tuberculosis, WBC and CRP (all P < 0.05), with interaction P values consistently greater than 0.05. Only sputum culture status showed a significant interaction (P < 0.001). The sputum-positive group exhibited a significantly higher risk of cavitation in patients with T2D (OR = 10.492, 95% CI: 3.266–33.711, P < 0.001), while the sputum-negative group showed a relatively lower risk (OR = 2.818, 95% CI: 1.280–6.207, P = 0.01). In contrast, none of the above stratification factors showed statistically significant associations with the risk of pulmonary cavitation in the NT2D group (all P>0.05), see **Table 7** and **Figure 4**.

**Table 7:** Subgroup analysis and interaction effect of T2D on pulmonary cavitation.

Items	OR	95%CI	P	Interaction P	NT2D OR (95%CI) P
Age				0.295	
<50	10.17	3.14, 33.02	<0.001^***^		Ref
≥50	3.28	1.54, 6.99	0.002^**^		1.539 (0.466, 5.089) 0.479
CD4+ T Cell (cells/μL)				0.676	
<200	4.7	2.17, 10.20	<0.001^***^		Ref
≥200	4.3	1.26, 14.74	0.02^*^		1.539 (0.466, 5.089) 0.479
HIV viral load (copies/mL)				0.535	
<50	4.5	1.25, 16.22	0.021^*^		Ref
≥50	5.23	2.22, 12.34	<0.001^***^		1.042 (0.294, 3.688) 0.950
ART				0.801	
Yes	4.58	1.97, 10.68	<0.001^***^		0.889 (0.301, 2.624) 0.831
No	4.81	1.81, 12.74	<0.001^***^		Ref
HIV				0.939	
Yes	4.82	2.57, 9.04	<0.001^***^		0.943 (0.394, 2.259) 0.895
No	4.52	1.64, 12.43	0.003^**^		Ref
Sputum Postive				<0.001^***^	
Yes	10.49	3.27, 33.71	<0.001^***^		1.921 (0.606, 6.097) 0.268
No	2.82	1.28, 6.21	0.01^*^		Ref
Distribution				0.812	
Intrapulmonary	4.96	2.30, 10.70	<0.001^***^		Ref
Intra- and extra-pulmonary	4.66	1.56, 13.90	0.006^**^		0.921 (0.299, 2.841) 0.886
WBC (×10^9^/L)				0.393	
<5.6	4.85	1.81, 13.02	0.002^**^		Ref
≥5.6	4.94	2.16, 11.30	<0.001^***^		1.883 (0.642, 5.524) 0.249
CRP (mg/L)				0.192	
<46.7	4.29	1.59, 11.62	0.004^**^		Ref
≥46.7	5.3	2.32, 12.11	<0.001^***^		1.918 (0.653, 5.631) 0.236
Current smoking status				0.337	
Smoker	5.11	2.414, 10.818	<0.001^***^		1.859 (0.564, 6.126) 0.308
Non-smoker	4.714	1.436, 15.479	0.011^*^		Ref

T2D, Type 2 diabetes mellitus; NT2D, Non-type 2 diabetes mellitus; OR, Odds ratio; CI, Confidence interval; ART, Antiretroviral therapy; WBC, White blood cell count; CRP, C-reactive protein; TB, Tuberculosis; Most subgroups analyzed in HIV-TB coinfected men; only the HIV status subgroup includes non-HIV TB men; interaction P < 0.05 indicates significant modifying effect of the stratification variable; * indicates P < 0.05, ** indicates P < 0.01, *** indicates P < 0.001; In the NT2D group, each stratification factor was treated as the core independent variable (e.g., sputum positivity vs. negativity, age < 50 years vs. ≥ 50 years) to analyze its association with pulmonary cavitation.

As shown in **Table 8**, stratified analyses according to glycemic control status showed distinct patterns in the associations between glycemic indicators and size of cavity. In the well-controlled glycemia subgroup, neither HbA1c nor FPG was significantly associated with size of cavity (all P > 0.05). In contrast, in the poorly controlled glycemia subgroup, both HbA1c (B = 1.193, 95% CI: 0.200–2.185, P = 0.02) and FPG (B = 0.852, 95% CI: 0.409–1.294, P < 0.001) were positively associated with increased size of cavity.

**Table 8:** Subgroup analysis of glycemic control status: linear regression analysis of continuous variables between HbA1c, FPG and pulmonary cavity size.

Items	HbA1c B (95%CI) P	FPG B (95%CI) P
Glycemic control status		
Well controlled	1.000 (-3.576, 5.576) 0.599	3.889 (-13.705, 21.483) 0.594
Poorly controlled	1.193 (0.200, 2.185) 0.020	0.852 (0.409, 1.294) <0.001

HbA1c, glycated hemoglobin; FPG, fasting plasma glucose; CI, confidence interval; Statistical method: Linear regression analysis was used to assess the association between continuous variables (HbA1c, FPG) and cavity size (continuous dependent variable). The B value represents the average change in cavity size (unit: mm) corresponding to a 1-unit increase in HbA1c (1%) or FPG (1 mmol/L).

## Discussion

Pulmonary cavitation remains a major clinical challenge in the management of HIV–TB coinfection, particularly among men, as it is closely associated with treatment failure, relapse, and ongoing transmission (20–22). Identifying modifiable risk factors for cavitation is therefore of substantial clinical importance. In this Comparative cross-sectional study of men with HIV–TB coinfection, we demonstrate that T2D is a strong risk factor for pulmonary cavitation. After comprehensive adjustment for immunologic, inflammatory, microbiological, and treatment-related factors, T2D was associated with nearly a fourfold increase in cavitation risk. In addition, poorer glycemic control, reflected by higher HbA1c and FPG levels, was linearly associated with larger cavity size.

These findings are consistent with and extend prior evidence linking hyperglycemia to more severe pulmonary TB manifestations. Previous studies in general TB populations have shown that patients with T2D are more likely to develop pulmonary cavities and extensive lung destruction (23, 24). Senlin Zhan et al. confirmed that individuals with elevated HbA1c or FPG levels were more likely to develop multiple lung cavities measuring at least 4 cm in diameter, which were typically associated with lobar involvement (25). Min Kyung Jung et al. also reported that patients with poorly controlled TB frequently exhibited imaging findings associated with cavitation risk factors, such as bronchial erosion and bronchiectasis, implying that hyperglycemia may promote cavitation by compromising bronchial wall integrity (26). However, current research on the association between T2D and pulmonary cavitation in HIV-TB co-infected patients remains limited. Although prior research have demonstrated consistent trends, the reported risk intensity of cavitation was lower than that in this study. This difference may be owing to the combined effect of HIV-induced immune suppression and hyperglycemia-mediated decrease of neutrophil bactericidal activity, which increases the likelihood of pulmonary cavitation in TB (4, 27).

To determine whether HIV infection modifies the observed association between T2D and pulmonary cavitation, we validated our findings in a non-HIV TB cohort using the same analytical framework. In this population, T2D remained significantly associated with cavitation risk, and higher FPG levels were correlated with larger cavity size. Although HbA1c was not significantly associated with cavity size in the non-HIV cohort, this discrepancy may reflect differences in immune status and inflammatory dynamics between HIV-infected and non-infected individuals. HbA1c represents long-term glycemic exposure, whereas acute TB-related inflammation in immunocompetent hosts may exert a stronger influence on short-term radiologic manifestations (28). Importantly, combined analyses across HIV-positive and HIV-negative populations confirmed that HIV status did not modify the core effect of T2D on pulmonary cavitation.

A key finding of this study is the significant interaction between T2D and sputum positivity. Among sputum-positive patients, T2D was associated with an exceptionally high risk of pulmonary cavitation, whereas the association was attenuated but still significant in sputum-negative patients. Sputum positivity generally reflects a higher mycobacterial burden, and when combined with hyperglycemia, may synergistically exacerbate pulmonary inflammation, impair bacterial clearance, and promote tissue destruction (29, 30). Consistent with this hypothesis, stratified analyses demonstrated that glycemic indicators were associated with cavity size only among patients with poorly controlled glycemia, but not among those with adequate glycemic control. These findings suggest that sustained hyperglycemia plays a critical role not only in cavitation occurrence but also in cavitary severity. These findings have important clinical implications since they show that sputum-positive T2D patients (especially poorly controlled glycemia) are at exceedingly high risk among HIV-TB co-infected persons. Among this group of people, to prevent the creation and progression of pulmonary tuberculosis cavities, improved measures are necessary, such as earlier beginning of anti-TB therapy, longer treatment duration, and more frequent CT surveillance.

Based on the core findings from the aforementioned subgroup analysis (T2D combined with sputum bacterial positivity is a significant risk factor for pulmonary cavitation), we assessed the predictive efficiency of several indicator combinations by ROC curve analysis (**Supplementary Table S6**). The results indicated that the integrated model of “T2D status + HbA1c + fasting blood glucose + sputum status” displayed robust predictive efficacy, achieving an AUC value of 0.813 (95% confidence interval: 0.741–0.886). This discovery indicates that the previously indicated clinical indications could facilitate swift risk classification for pulmonary cavitation in male patients co-infected with HIV and tuberculosis in clinical settings. These findings should be interpreted as exploratory and hypothesis-generating rather than definitive predictive tools.

Notably, CD4^+^ T-cell counts were higher in the T2D group than in the NT2D group, a finding that contrasts with some prior reports (31–33). To clarify this discrepancy, we performed CD4-stratified analyses (**Supplementary Table S7**). Following stratification, no significant connection between T2D and CD4+ T-cell counts was seen in either the low- or high-CD4 subgroups, implying that the crude between-group difference may represent Simpson’s paradox or variability in comorbidities among study populations (34). More importantly, the association between T2D and pulmonary cavitation remained consistent across CD4^+^ strata, indicating that the observed effect of T2D is not driven by differences in immune status as measured by CD4^+^ T-cell counts.

Furthermore, the lower ART uptake but longer ART duration observed in the T2D group may reflect differences in disease course and treatment sequencing among patients with multiple comorbidities. Supplementary stratified analyses among ART-treated individuals showed that patients with T2D had longer durations of HIV infection and ART exposure, which is consistent with clinical practice patterns and does not undermine the robustness of the primary findings (**Supplementary Table S8**). Importantly, adjustment for ART status in multivariable models ensured that ART-related factors did not confound the association between T2D and pulmonary cavitation.

One of the most practical ramifications of our research is the inclusion of T2D-related variables in the risk assessment of pulmonary cavitation in HIV and tuberculosis patients. Historically, risk assessment for pulmonary cavitation in these patients has relied solely on CD4+ cell counts, HIV viral load, and ART efficiency, sometimes overlooking metabolic variables (28). This omission may underestimate the risk of pulmonary cavitation in patients with T2D and moderate CD4+ levels, thereby misclassifying them as low-risk and missing out on increased therapies.

Our study has several advantages: Firstly, it focuses primarily on male patients with the triple comorbidity of HIV, TB and T2D, thus partially filling a gap in previous research. The study controlled for confounding factors in regression analyses and validated the robustness of the findings through subgroup analyses and interaction tests. Furthermore, the study explored the association between T2D and pulmonary cavitation and quantified the linear relationship between glycemic indicators and cavity size. Additionally, the study identified a potential synergistic effect between sputum smear positivity and hyperglycaemia, which could significantly increase the risk of pulmonary cavitation.

However, we must acknowledge the limitations of this study. First, the center-based study design restricted the generalizability of the results, and the retrospective data collection method limited the inference of causal relationships. Second, detailed obesity measures and complete data on some comorbidities were unavailable, which may introduce residual confounding and should be considered when interpreting the results. Third, certain variables, such as specific ART regimens and Mycobacterium tuberculosis load, were unavailable and may further introduce confounding bias. Furthermore, although sensitivity analyses suggested that pulmonary TB diagnostic methods had little impact on the association between T2D and pulmonary cavitation, differences in detection efficacy among diagnostic approaches cannot be fully eliminated. Future studies should adopt a unified, highly sensitive detection tool. In addition, this study is limited by including only men with HIV-TB coinfection. While men are at higher risk and tend to develop cavities faster, the findings may not apply to women, and it remains unclear whether T2D’s impact on pulmonary cavities is gender-specific. Finally, due to the lack of routine testing for mycobacterial load, surrogate indicators such as sputum smear status, cavity occurrence, and cavity size were used, which may introduce residual confounding.

Future research should conduct prospective, multicenter validation and investigate mechanisms using *in vitro*/*in vivo* tests and animal models, and meanwhile prioritize addressing the research gap in gender differences. Clinically, including critical parameters such as glycemic markers, sputum culture results, and CD4+ counts into a pulmonary cavitation risk prediction tool tailored to this cohort may allow for better disease treatment.

## Conclusion

T2D and elevated glycemic levels are strongly associated with pulmonary cavitation in men with HIV–tuberculosis coinfection. These associations remain robust after adjustment for immunologic, inflammatory, and treatment-related factors and are not modified by HIV status. Poor glycemic control is linked to greater cavitary burden, particularly among sputum-positive patients, identifying a subgroup at exceptionally high risk. Integrating routine glycemic evaluation and optimized diabetes management into the clinical care of HIV–TB patients may improve risk stratification and support targeted interventions to mitigate cavitation-related disease severity.

## Data Availability

The raw data supporting the conclusions of this article will be made available by the authors, without undue reservation.
