# Supplementary material for: Type 2 diabetes is associated with pulmonary cavitation in men with HIV-TB coinfection
Source: Front Endocrinol (Lausanne). 2026 Jan 23;17:1635725. doi: 10.3389/fendo.2026.1635725 (PMC12875991; doi:10.3389/fendo.2026.1635725)
Supplement: Supplementary file 1 [file DataSheet1.docx]

**1.** **Supplementary Table S1 Description**

Supplementary Table S1 compares the baseline clinical, metabolic, and laboratory characteristics between HIV-positive and non-HIV individuals to verify the comparability of the two cohorts (used for validating core findings).

Key observations:

Demographic and TB related core indicators showed no significant intergroup differences: age (55.27±12.08 vs. 53.36±12.74 years, P=0.237), pulmonary cavitation rate (26.2% vs. 21.0%, P=0.342), sputum positivity rate (27.0% vs. 22.0%, P=0.35), TB distribution (intrapulmonary vs. intra- and extra-pulmonary, P=0.91), cavity size (17.57 vs. 14.00 mm, P=0.24), and WBC count (6.31 vs. 5.67, P=0.52).

Metabolic and inflammatory indicators exhibited expected differences consistent with cohort characteristics: HIV-positive individuals had a higher proportion of type 2 diabetes mellitus (T2DM, 49.8% vs. 30.0%, P<0.001), as well as higher glycated hemoglobin (HbA1c, 9.20 vs. 6.50, P<0.001), fasting plasma glucose (FPG, 11.36 vs. 7.71, P<0.001), and C-reactive protein (CRP, 64.45 vs. 15.09, P<0.001). These differences reflect the inherent metabolic and inflammatory features of the HIV-TB-T2DM syndemic population and do not affect the comparability of core TB-related outcomes.

In summary, the two cohorts are well comparable in key baseline characteristics related to TB and pulmonary cavitation, confirming the validity of using the non-HIV cohort as a validation group for the study’s core findings.

**Table S1 Baseline characteristics comparison between HIV-positive and non-HIV individuals**

| Items | HIV | Non-HIV | P |
| --- | --- | --- | --- |
| Age | 55.27±12.08 | 53.36±12.74 | 0.237 |
| T2D |  |  | <0.001*** |
| Yes | 132 | 30 |  |
| No | 131 | 70 |  |
| Cavities |  |  | 0.342 |
| Yes | 69 | 21 |  |
| No | 194 | 79 |  |
| Sputum Postive |  |  | 0.35 |
| Yes | 71 | 22 |  |
| No | 192 | 78 |  |
| Distribution |  |  | 0.91 |
| Intrapulmonary | 170 | 64 |  |
| Intra- and extra-pulmonary | 93 | 36 |  |
| HbA1c | 9.20 (7.10,12.80) | 6.50 (5.95, 10.00) | <0.001*** |
| FPG | 11.36 (8.13, 16.80) | 7.71 (4.89, 9.11) | <0.001*** |
| WBC | 6.31 (4.70, 8.25) | 5.67 (4.83, 10.62) | 0.52 |
| CRP | 64.45 (32.90, 104.20) | 15.09 (4.15, 40.64) | <0.001*** |
| Size of cavity (mm) | 17.57 (13.21, 31.00) | 14.00 (11.52, 15.14) | 0.24 |

Note: T2D = Type 2 diabetes mellitus; NT2D = Non-type 2 diabetes mellitus; HbA1c = Glycated hemoglobin A1c; FPG = Fasting plasma glucose; WBC = White blood cell count; CRP = C-reactive protein; * indicates P < 0.05, ** indicates P < 0.01, *** indicates P < 0.001; b denotes the use of the Mann-Whitney U test, c denotes the use of Chi-square test,

**2. Supplementary Table S2**

**Table S2 Laboratory Instruments and Corresponding Reagents**

| Category | Parameter | Method | Instrument | Reagent (Lot No.) |
| --- | --- | --- | --- | --- |
| Metabolic Parameters | HbA1c | Ion-exchange HPLC | Bio-Rad D-10 | Bio-Rad HbA1c Reagent Kit (230425) |
|  | FPG | Hexokinase assay | Beckman Coulter AU5800 | Beckman Coulter Glucose Reagent (20230518) |
| Hematological Profiles | WBC/RBC/PLT | Automated analysis | Sysmex XN-9100 | Sysmex XN-Control Materials (230601) |
|  | CRP | Immunoturbidimetric assay | Siemens BNII | Siemens N Latex CRP Reagent (230312) |
|  | IL-6 | Chemiluminescence immunoassay | Roche Cobas e601 | Roche IL-6 Reagent Kit (230518) |
| Lymphocyte Subsets | CD3⁺/CD4⁺/CD8⁺ T cells | Four-color flow cytometry | BD FACSCanto II | BD Multitest TBNK Reagent (230612); BD TruCOUNT™ Tubes (230409) |
|  | LYC | Calculated from differential counts (hematology analyzer) + TruCOUNT™ beads calibration | Sysmex XN-9100 + BD FACSCanto II | Sysmex XN-Control Materials (230601); BD TruCOUNT™ Tubes (230409) |
| TB-Related Tests | Sputum staining | Ziehl-Neelsen staining | - | Sigma-Aldrich Ziehl-Neelsen Stain Kit (230309) |
|  | MTB culture | MGIT/LJ culture | BD BACTEC MGIT 960 | BD MGIT Tubes (230218); LJ Medium (230215) |
| HIV-Related Tests | HIV viral load | Real-time RT-PCR | Abbott m2000rt | Abbott HIV-1 Viral Load Assay (230525) |

**3. Supplementary Table S3, S4 Description**

Regarding the reviewer's concern that "the detection rates of the three diagnostic methods—microscopy, LJ culture, and MGIT culture may affect the study results,“ this study employed dual validation through stratified analysis (Table S3) and model adjustment (Table S4). This systematic evaluation of diagnostic method differences on the ”association between T2DM and pulmonary cavities" ensures the robustness of the core conclusions.

**(1) Stratified Analysis by Diagnostic Method (Table S3)**

Table S3 stratifies data according to three core diagnostic methods for pulmonary tuberculosis (sputum smear microscopy, LJ culture, MGIT culture) to separately examine the strength of association between T2D and pulmonary cavitation within each diagnostic subgroup. This directly addresses the concern regarding whether diagnostic methods with differing detection rates may confound results.

The results showed:

Subgroup with positive sputum smear microscopy (n = 71): T2DM was significantly associated with an increased risk of pulmonary cavitation (OR = 10.492, 95% CI = 3.266–33.711, P < 0.001), indicating a clear effect of T2DM on the risk of pulmonary cavitation under this diagnostic criterion.

LJ culture-positive subgroup (n = 155): T2DM remained significantly associated with pulmonary cavitation (OR = 3.901, 95% CI = 1.435–10.605, P = 0.008). The direction of the association was consistent with that observed in the subgroup with a positive sputum smear microscopy result, though the strength of the association differed slightly due to the varying detection rates of the two methods. The core association remained unchanged.

MGIT culture-positive subgroup (n = 37): No statistically significant association was observed (OR = 1.400, 95% CI = 0.340–5.765, P = 0.641). This is primarily due to the small sample size in this subgroup, which resulted in insufficient statistical power.

**Table S3 Association between T2DM and pulmonary cavitation stratified by pulmonary tuberculosis diagnostic methods**

| Diagnostic Methods for Pulmonary Tuberculosis | n | OR (95CI%) P |
| --- | --- | --- |
| Sputum postive | 71 | 10.492 (3.266, 33.711) <0.001 |
| LJ culture positive | 155 | 3.901 (1.435, 10.605) 0.008 |
| MGIT culture positive | 37 | 1.400 (0.340, 5.765) 0.641 |

Note: TB = Tuberculosis; LJ = Löwenstein-Jensen (culture medium); MGIT = Mycobacteria Growth Indicator Tube (automated culture system); OR = Odds ratio; CI = Confidence interval.

**(2) Robustness Validation After Adjusting Diagnostic Methods (Table S4)**

Table S4 further quantifies the potential confounding effects of diagnostic methods on core associations by comparing the “unadjusted original model” with the “adjusted model incorporating diagnostic methods as covariates,” thereby addressing limitations in the stratified analysis of the MGIT subgroup.

The results showed:

Original model (unadjusted for diagnostic method): The OR for T2D associated with pulmonary cavities was 4.822 (95% confidence interval (CI)=2.573–9.037, P<0.001).

Adjusted model (incorporating the three diagnostic methods as dummy variables to control for differences in detection rates): The OR value fluctuated slightly to 4.565 (95% CI = 2.411–8.646, P < 0.001), a fluctuation of only 5.3%, and remained statistically significant.

This clearly indicates that, even when accounting for differences in detection rates among the three diagnostic methods, their confounding effect on the association between T2DM and pulmonary cavities is minimal.

**Table S4 Robustness verification of the T2DM-pulmonary cavitation association after adjusting for pulmonary tuberculosis diagnostic methods**

| Model | OR (95CI%) P |
| --- | --- |
| Original model | 4.822 (2.573, 0.037) <0.001 |
| Adjusted model | 4.565 (2.411, 8.646) <0.001 |

Note: Original model: unadjusted for TB diagnostic methods; Adjusted model: adjusted for pulmonary TB diagnostic methods as covariables to quantify potential confounding effects.

**4.** **Definition and Measurement Methods of Disease Duration, Comorbidity Assessment**

（1）Disease duration:

1)Duration of HIV infection: Calculated as the number of days from the date of HIV diagnosis to the date of admission for the current pulmonary tuberculosis diagnosis.

2)Duration of ART use: Calculated as the number of days from the date of initial ART initiation to the date of the current admission.

3)Duration of TB disease: Calculated as the number of days from the date of first occurrence of pulmonary tuberculosis symptoms or the date of TB diagnosis to the date of the current admission.

4)Duration of T2D: Calculated as the number of days from the date of T2D diagnosis to the date of the current admission.

5)Duration of diabetes treatment: Calculated as the number of days from the date of initial initiation of glucose-lowering therapy (including oral hypoglycemic agents or insulin therapy) to the date of the current admission.

（2）Comorbidity Assessment:

1)Hypertension: Defined per the Chinese Guidelines for the Management of Hypertension (2024), i.e., systolic blood pressure ≥140 mmHg and/or diastolic blood pressure ≥90 mmHg, or prior diagnosis with ongoing antihypertensive treatment(1).

2)Chronic Obstructive Pulmonary Disease (COPD): Diagnosed per the Guidelines for the Diagnosis and Treatment of Chronic Obstructive Pulmonary Disease (2021), combining clinical symptoms (chronic cough, sputum production, dyspnea), pulmonary function tests (FEV1/FVC <70%), and chest imaging findings(2).

3)Fatty liver: Confirmed by abdominal ultrasound (typical manifestations: enhanced liver parenchymal echo, unclear intrahepatic duct structure), excluding alcoholic liver disease, viral hepatitis, and other liver disorders.

4)Hyperlipidemia: Defined per the Chinese Guidelines for the Prevention and Treatment of Dyslipidemia in Adults (2023), i.e., total cholesterol ≥5.2 mmol/L, triglycerides ≥1.7 mmol/L, low-density lipoprotein cholesterol ≥3.4 mmol/L, or high-density lipoprotein cholesterol <1.0 mmol/L, or prior diagnosis with ongoing lipid-lowering treatment(3).

5)Viral hepatitis: Diagnosed based on positive hepatitis virus markers (e.g., HAV IgM antibody, HBsAg, HCV antibody) combined with clinical diagnosis.

**5. Supplementary Table S5**

Table S5 summarizes the hypoglycemic medications and treatment patterns among patients with T2D in the study cohort. Oral hypoglycemic agents (OHAs) and insulin preparations are presented according to their pharmacological classes and specific drug names as recorded in medical records. Percentages were calculated based on the number of patients receiving hypoglycemic treatment. Treatment patterns were categorized as monotherapy with OHAs, combination therapy with OHAs, monotherapy with insulin, or combination therapy with insulin and OHAs. Patients for whom detailed hypoglycemic regimens were unavailable were classified as having an unknown hypoglycemic regimen.

**Table S5 Summary Table of Medication-Related Information in Patients with T2D**

| Medication-Related Information | Specific Category | Specific Medication | Number of Users | Percentage of Treated Patients |
| --- | --- | --- | --- | --- |
| OHAs | Biguanides | Metformin | 39 | 50.65% |
|  | Sulfonylureas | Gliclazide | 11 | 13.29% |
|  |  | Glimepiride | 5 | 6.49% |
|  |  | Gliquidone | 1 | 1.30% |
|  | Meglitinides | Repaglinide | 5 | 6.49% |
|  | α-Glucosidase Inhibitors | Acarbose | 8 | 10.39% |
|  |  | Voglibose | 1 | 1.30% |
|  | SGLT-2 Inhibitors | Dapagliflozin | 1 | 1.30% |
| Insulin | Short-acting Insulin | Yousiling Insulin | 1 | 1.30% |
|  |  | Humulin Insulin | 3 | 3.90% |
|  |  | Novolin Insulin | 1 | 1.30% |
|  |  | Gansulin Insulin | 1 | 1.30% |
|  | Intermediate-acting Insulin | Isophane Insulin | 3 | 3.90% |
|  | Long-acting Insulin | Glargine Insulin | 4 | 5.19% |
| Unknown Hypoglycemic Regimen | - | - | 13 | 16.88% |
| Monotherapy with OHAs | - | - | 32 | 42.00% |
| Combination Therapy with OHAs | - | - | 20 | 25.97% |
| Monotherapy with Insulin | - | - | 19 | 24.68% |
| Combination Therapy (Insulin + OHAs) | - | - | 2 | 2.60% |

Note: Percentages were calculated based on the number of patients receiving hypoglycemic treatment.

**6. Supplementary Table S6 Description**

Supplementary Table S6 aims to systematically evaluate the predictive value of different combinations of metabolic indicators (T2D status, HbA1c, FPG) and sputum bacteriology status for pulmonary cavitation in HIV-TB male patients, thereby identifying clinically practical risk screening strategies.

Results showed:

Significant differences in predictive performance among individual indicators: T2D status alone demonstrated no statistically significant predictive value (AUC=0.569, 95% CI=0.476–0.662, P=0.164), with a specificity of only 11.4%, indicating limited clinical utility; Sputum bacterial status (AUC=0.709, P<0.001) and metabolic indicator combinations (HbA1c+FPG, AUC=0.723, P<0.001) demonstrated moderate predictive performance. Sputum bacterial status achieved 83.7% specificity, suitable for preliminary exclusion of low-risk individuals.

Combining multiple indicators significantly enhanced predictive performance: the three-marker combination of “T2D status + HbA1c + FPG” increased AUC to 0.738 (P<0.001), representing a 1.5% improvement over dual metabolic markers. Further inclusion of sputum bacterial status yielded the optimal four-marker combination (T2D status + HbA1c + FPG + sputum bacterial status), achieving AUC=0.813 (95% CI=0.741–0.886, P<0.001), sensitivity 65.5%, and specificity 87.0%, meeting criteria for a clinically good predictive model.

Core Value: This optimal combination comprises only four routine clinical indicators with remarkable specificity of 87.0%. This finding suggests clinicians could rapidly identify low-risk patients for pulmonary cavitation using this model, thereby reducing unnecessary chest CT follow-ups. Simultaneously, for patients classified as high-risk by the model (especially those with sputum bacteria positivity and elevated blood glucose), prioritizing enhanced glycemic control and anti-TB interventions could improve diagnostic and therapeutic precision.

In summary, ROC curve analysis confirms that the multidimensional combination of “metabolic indicators + sputum bacterial status” represents the optimal approach for predicting pulmonary cavitation in HIV-TB male patients. This not only validates the robustness of the core conclusions presented in the main text but also provides a directly implementable clinical screening tool, significantly enhancing the translational value of the research.

**Table S6 Comparison of ROC curve predictive performance of different predictive index combinations for pulmonary cavitation in HIV-TB coinfected men**

| Items | AUC (95%CI) | P | Optimal Cut-off | | Sensitivity (%) | | Specificity (%) | |
| --- | --- | --- | --- | --- | --- | --- | --- | --- |
| T2D | 0.569 (0.476, 0.662) | 0.164 | 0.138 | 96.4 | | 11.4 | |  |
| HbA1c+FPG | 0.723 (0.638, 0.807) | <0.001^***^ | 0.34 | 76.4 | | 57.4 | |  |
| Sputum Postive | 0.709 (0.619, 0.800) | <0.001^***^ | 0.419 | 58.2 | | 83.7 | |  |
| T2D+HbA1c+FPG | 0.738 (0.655, 0.821) | <0.001^***^ | 0.384 | 76.4 | | 62 | |  |
| T2D+HbA1c+FPG+Sputum Postive | 0.813 (0.741, 0.886) | <0.001^***^ | 0.525 | 65.5 | | 87 | |  |

Note: T2D = Type 2 diabetes mellitus; NT2D = Non-type 2 diabetes mellitus; HbA1c = Glycated hemoglobin A1c; FPG = Fasting plasma glucose; The optimal cut-off values were determined by maximizing the Youden index (sensitivity + specificity - 1); * indicates P < 0.05, ** indicates P < 0.01, *** indicates P < 0.001.

**7. Supplementary Table S7 Description**

Supplementary Table 4 uses stratified analysis based on CD4+ T cell counts to investigate whether the association between T2DM and pulmonary cavities is influenced by immune status (CD4+ levels). The analysis also seeks to explain the apparent paradox of 'gross differences in CD4+ counts between the T2DM group and the NT2D group', thereby validating the robustness of the core findings.

The results indicate:

Within each CD4+ stratified subgroup, no statistically significant differences in CD4+ T cell counts were observed between T2DM and NT2D patients.

In the CD4+ <200 cells/μL subgroup, the T2DM group had a CD4+ count of 83.00 (30.00, 122.75) cells/μL, while the NT2D group had 63.00 (29.00, 110.25) cells/μL (P = 0.495). - In the CD4+ ≥200 cells/μL subgroup, the T2DM group had a count of 307.00 (237.50, 361.00) cells/μL, while the NT2D group had a count of 374.50 (238.75, 439.75) cells/μL (P = 0.171). This suggests that the previously observed differences in CD4+ counts between the two groups may be due to Simpson's paradox or comorbidity heterogeneity, as no substantive differences were observed after stratification.

Key association robustness: T2DM was significantly associated with an increased risk of pulmonary cavitation regardless of the level of CD4+ T cells. In the CD4+ <200 cells/μL subgroup, the adjusted odds ratio (OR) was 4.895 (95% confidence interval (CI)=2.238–10.706, P<0.001), and in the CD4+ ≥200 cells/μL subgroup, the adjusted OR was 3.643 (95% CI=1.034–12.843, P=0.044).

**Table S6 Association between T2DM and pulmonary cavitation stratified by CD4+ T cell count (robustness verification)**

| CD4+ Subgroup | Subgroup Type | CD4+ T Cell (cells/μL) M (Q1, Q3) | P | Adjusted Model OR (95%CI) P |
| --- | --- | --- | --- | --- |
| <200 | T2D | 83.00 (30.00,122.75) | 0.495 | 4.895 (2.238, 10.706) <0.001 |
|  | NT2D | 63.00 (29.00, 110.25) |  |  |
| ≥200 | T2D | 307.00 (237.50, 361.00) | 0.171 | 3.643 (1.034, 12.843) 0.044 |
|  | NT2D | 374.50 (238.75, 439.75) |  |  |

Note: T2D = Type 2 diabetes mellitus; NT2D = Non-type 2 diabetes mellitus; The adjusted model accounted for potential confounding factors (except for the exclusion of CD4+ count, the rest align with the regression model in the HIV+ population described in the main text).

**8.Supplementary Table S8 Description**

To validate the differences in ART-related indicators between the T2D group and the NT2D group reported in the baseline characteristics (i.e., lower ART coverage but longer duration in the T2D group), this supplementary analysis focused on patients currently receiving ART (58 in the T2D group, 43.9% of the total T2D population; 85 in the NT2D group, 67.2% of the total NT2D population). The Wilcoxon rank-sum test (two-sided, α = 0.05) was used to compare HIV infection duration and ART duration between groups, both of which were skewed and are presented as median (first quartile, third quartile) [M (Q1, Q3)]. Results showed that the median HIV infection duration in the T2D group was 730.00 days (Q1 = 75.00 days, Q3 = 2007.00 days), significantly longer than 210.00 days (Q1 = 30.00 days, Q3 = 730.00 days) in the NT2D group (Z = -3.128, P = 0.002). The median ART duration in the T2D group was 728.00 days (Q1 = 44.00 days, Q3 = 1799.00 days), significantly longer than 90.00 days (Q1 = 16.50 days, Q3 = 452.50 days) in the NT2D group (Z = -3.305, P < 0.001). This finding is consistent with the clinical reality of HIV–TB–T2D coinfected patients: due to comorbid diabetes, active tuberculosis, and immune deficiency, multiple organs are often involved, and there is a high risk of drug–drug interactions. Clinicians commonly adopt individualized ART initiation strategies, and those who have started ART are typically long-term HIV patients with more stable disease and higher treatment adherence. This supplementary analysis was conducted solely to verify the logical consistency of the main text data; the independence of the core conclusion—i.e., the association between T2D and pulmonary cavitation—has already been confirmed through multivariable analyses in the main text (adjusting for ART status).

**Table S7 Comparison of HIV Infection Duration and ART Duration Among ART-Positive Patients [T2D (n=58 vs. NT2D n=85]**

| Items | T2D [M (Q1, Q3)] | NT2D [M (Q1, Q3)] | Z | P |
| --- | --- | --- | --- | --- |
| Duration of HIV infection (days) | 730.00 (75.00, 2007.00) | 210.00 (30.00, 730.00) | -3.128 | 0.002 |
| Duration of ART use (days) | 728.00 (44.00, 1799.00) | 90.00 (16.50, 452.50) | -3.305 | <0.001 |

**REFERENCES**

1. Clinical practice guideline for the management of hypertension in China. Chin Med J (Engl) (2024) 137:2907–2952. doi: 10.1097/CM9.0000000000003431

2. [guidelines for the diagnosis and management of chronic obstructive pulmonary disease (revised version 2021)]. Zhonghua jie he he hu xi za zhi = Zhonghua jiehe he huxi zazhi = Chin j tuberc respir dis (2021) 44:170–205. doi: 10.3760/cma.j.cn112147-20210109-00031

3. Li J-J, Zhao S-P, Zhao D, Lu G-P, Peng D-Q, Liu J, Chen Z-Y, Guo Y-L, Wu N-Q, Yan S-K, et al. 2023 chinese guideline for lipid management. Front Pharmacol (2023) 14:1190934. doi: 10.3389/fphar.2023.1190934
